# Supplementary material for: Assessing the impact of free-roaming dog population management through systems modelling
Source: Sci Rep. 2022 Jul 6;12:11452. doi: 10.1038/s41598-022-15049-1 (PMC9259565; doi:10.1038/s41598-022-15049-1)
Supplement: Supplementary file 1 — Supplementary Information. [file 41598_2022_15049_MOESM1_ESM.docx]

**Supplementary information**

**Description of initial state variables**

Initial sizes of dog populations were estimated for the baseline simulation, based on our previous research in Lviv ^[1]^. We extrapolated estimates of free-roaming dog population size to provide a city-wide estimate of approximately 14,000 dogs and inflated this estimate to an assumed initial population size of 20,000 dogs, to account for the ongoing population management in Lviv. The carrying capacity for free-roaming dogs in this urban environment depends on the availability of resources (i.e. food, shelter, water, and human attitudes and behaviour ^[2]^). The carrying capacity is challenging to estimate for the free-roaming dog populations. In this systems model, we estimated that the free-roaming dog population was at a carrying capacity of 20,000 dogs.

An initial shelter dog population size was estimated based on the five currently registered shelters in Lviv (VIER PFOTEN International, personal communication), providing an initial shelter dog population (*H*) of 3,750 dogs (approximately 750 dogs per shelter).

The initial owned dog population (*O*) was extrapolated from the dog ownership estimates based on our previous research in Ukraine^[3]^. The level of ownership reported in Ukraine was 56% and most owners had only one dog. In a city the size of Lviv (human population size: 717,803), and estimating approximately four people per household, this equated to an initial owned dog population (*O*) of 100,492 dogs. The owned dog population size was assumed to be at the carrying capacity (*K_o_*).

**Description of flows**

*Abandonment/roaming rate*

Estimating the rate at which owned dogs are abandoned (*α)* is difficult, as owners are likely to under-report abandonment of dogs. Our previous research in Ukraine using a questionnaire survey provided an indication of the rate of owned dog abandonment of between 0 and 0.0045 per dog over its lifetime ^[3]^. Given the likely under-reporting, and potential biases associated with questionnaire surveys, it can be assumed that the actual rate of abandonment is higher. Hsu, Severinghaus & Serpell (2003) reported a higher rate of abandonment of 0.05 per dog-owning lifetime in Taiwan ^[4]^, with a higher rate of 0.32 of respondents answering that they knew of someone who had abandoned a dog in their dog-owning lifetime ^[4]^. Fielding & Plumridge (2005) ^[5]^ in The Bahamas report a combined abandonment/killing rate of around 0.10 per year. If half of these dogs were abandoned and half were killed, this equates to approximately 0.004 abandonment rate per month. For simplicity, we combine the rate of abandonment with the rate at which owned dogs change from restricted to roaming (i.e. from the owned restricted subpopulation to the free-roaming dog population). Given there may be differences in abandonment rates between different countries, and the likely under-reporting by respondents in the questionnaire, we estimated a monthly abandonment/roaming rate of 0.003.

*Adoption rate of shelter dogs/Rehoming rate*

Shelter rehoming rates vary across shelters and countries, reported rates vary between 0.07 and 0.43 of the shelter population per year ^[6–12]^. Data from shelters in Lviv average at around 0.47 rehoming rate per year, equating to a rehoming rate of approximately 0.04 of the shelter dog population per month. Not all shelters within the city may be as successful. To account for this, we assumed a 0.025 rehoming rate (*β*) per month.

*Intrinsic growth rate of free-roaming dog population*

The intrinsic growth rate of a population (also referred to as the Malthusian parameter) describes the growing potential of a population. This rate can be estimated simply by births minus deaths ^[13,14]^, leading to estimates of 0.01 per month (^[13]^ estimated using parameters from free-roaming dog populations in the USA by ^[15]^) and 0.02 per month (^[14]^ estimated using parameters from free-roaming dog populations in Tanzania ^[16]^). Calculating the intrinsic growth rate using this simple calculation may not necessarily reflect the maximum intrinsic growth rate, instead reflecting the growth rate for a population under their current conditions ^[17]^. Maximum intrinsic growth rate can be estimated using fecundity and survivorship (e.g. Lotka equation ^[18–20]^). In terms of dog populations, Acosta-Jamett *et al.,* (2010) ^[21]^ estimated the intrinsic growth rate of 0.02 per month for an owned population of dogs in Chile. Intrinsic growth rates are population specific (i.e. different populations may have different maximum intrinsic growth rates).

*Growth rate of owned dogs*

As studies have reported a stable owned dog population over time ^[22]^, we assumed that demand for dogs was met quickly through a supply of dogs from owned dog births, breeders and friends. The maximum growth rate of owned dogs (*r_o_*) was therefore assumed to be higher than that of free-roaming dog growth rate (*r_s_*), at 0.07 per month.

*Relinquishment rate of owned dogs*

The rate of owned dog relinquishment was also difficult to estimate, as studies tended to report the percentage of the shelter dog population that have been relinquished each year, rather than how this equated to the percentage of the owned dog population. Our previous research suggested a relinquishment rate between 0.001 and 0.007 per dog-owning lifetime ^[3]^. New *et al.,* (2004) ^[23]^ conducted a survey on households in the USA and reported an annual relinquishment rate of 0.008. We assumed the rate of owned dog relinquishment (*γ*) was the same as reported by New *et al.,* (2004) ^[23]^ at 0.008 per year, equating to 0.0007 per month.

*Free-roaming dog adoption rate*

There was evidence from our previous research that some dog owners acquired their dogs from the free-roaming dog population (Bulgaria 35.5%, Italy 24.8%, and Ukraine 34.6%) ^[3]^. An average (across the studied countries) of 32% of owned dogs had been acquired from the free-roaming dog population. We assumed the free-roaming dog adoption rate (*δ*) was lower than the shelter adoption rate, at 0.007 per month.

*Shelter dog death rate*

In this simulation, we assumed shelters operated with a “no kill” policy (i.e. dogs were not killed in shelters as part of population management). The shelter dog death rate was included to incorporate deaths due to behavioural problems, health problems and natural mortality (e.g. due to age). We assumed that a shelter dog had a life expectancy of 10 years, slightly shorter than the life expectancy of owned dogs (between 12 to 14 years ^[24]^). This equates to a shelter dog death rate (*μ_h_*) of 0.008 per month.

*Neutered dog death rate after release*

Free-roaming dog death rates vary by age, sex, association with people (i.e. access to resources), and geographic location. Reported estimates vary between 0.15 and 0.52 per year, equating to approximately between 0.01 and 0.04 per month and a life expectancy of two to seven years ^[16,21,25,26]^. Using these reported estimates, we modelled neutered free-roaming dog death rate (*μ_n_*) explicitly for the CNR intervention at a minimum death rate of 0.02 per month. We modelled a density dependent death rate (i.e. when the population becomes closer to the carrying capacity the death rate is greater). The death rate was a non-linear function of population size and carrying capacity modelled using a table lookup function (Figure S1).


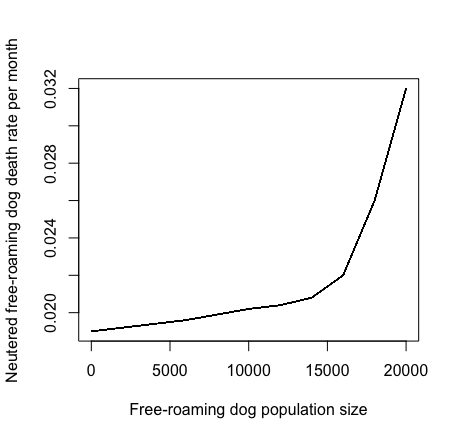


Figure S1. Neutered free-roaming dog death rate lookup function.

**Sensitivity Analyses**


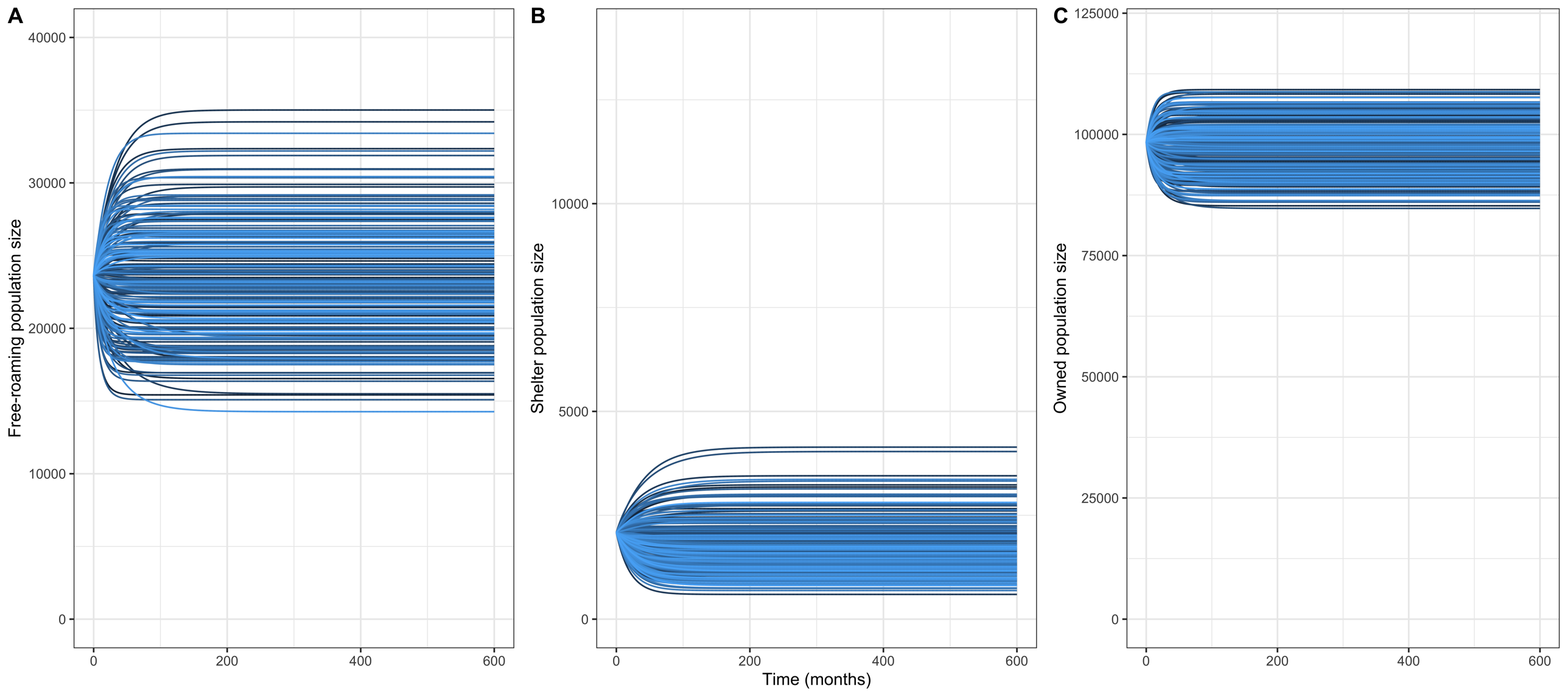


Figure S2. Results of global sensitivity analysis in **baseline simulation** on the (A) free-roaming, (B) shelter, and (C) owned dog population sizes.

Simulations generated 100 times and run for 600 months (50-years).





Figure S3. Local sensitivity analysis assessing the effect of parameters on the free-roaming, shelter and owned dog population equilibrium size. Parameters include: (i) carrying capacity of owned dogs (*K_o_*) on (A) free-roaming dog population, (B) shelter dog population, and (C) on owned dog population; (ii) carrying capacity of free-roaming dogs (*r_s_*) on (D) free-roaming dog population, (E) shelter dog population, and (F) on owned dog population; (iii) growth rate of owned dogs (*r_o_*) on (G) free-roaming dog population, (H) shelter dog population, and (I) on owned dog population; and (iv) growth rate of free-roaming dogs (*r_s_*) on (J) free-roaming dog population, (K) shelter dog population, and (L) on owned dog population.





Figure S4. Local sensitivity analysis assessing the effect of parameters on the free-roaming, shelter and owned dog population equilibrium size. Parameters include: (i) abandonment rate (*α*) on (A) free-roaming dog population, (B) shelter dog population, and (C) on owned dog population; (ii) relinquishment rate (*γ*) on (D) free-roaming dog population, (E) shelter dog population, and (F) on owned dog population; (iii) shelter adoption rate (*β*) on (G) free-roaming dog population, (H) shelter dog population, and (I) on owned dog population; (iv) shelter death rate (*μ_h_*)on (J) free-roaming dog population, (K) shelter dog population, and (L) on owned dog population; and (v) free-roaming dog adoption rate (*δ*) on (M) free-roaming dog population, (N) shelter dog population, and (O) on owned dog population.


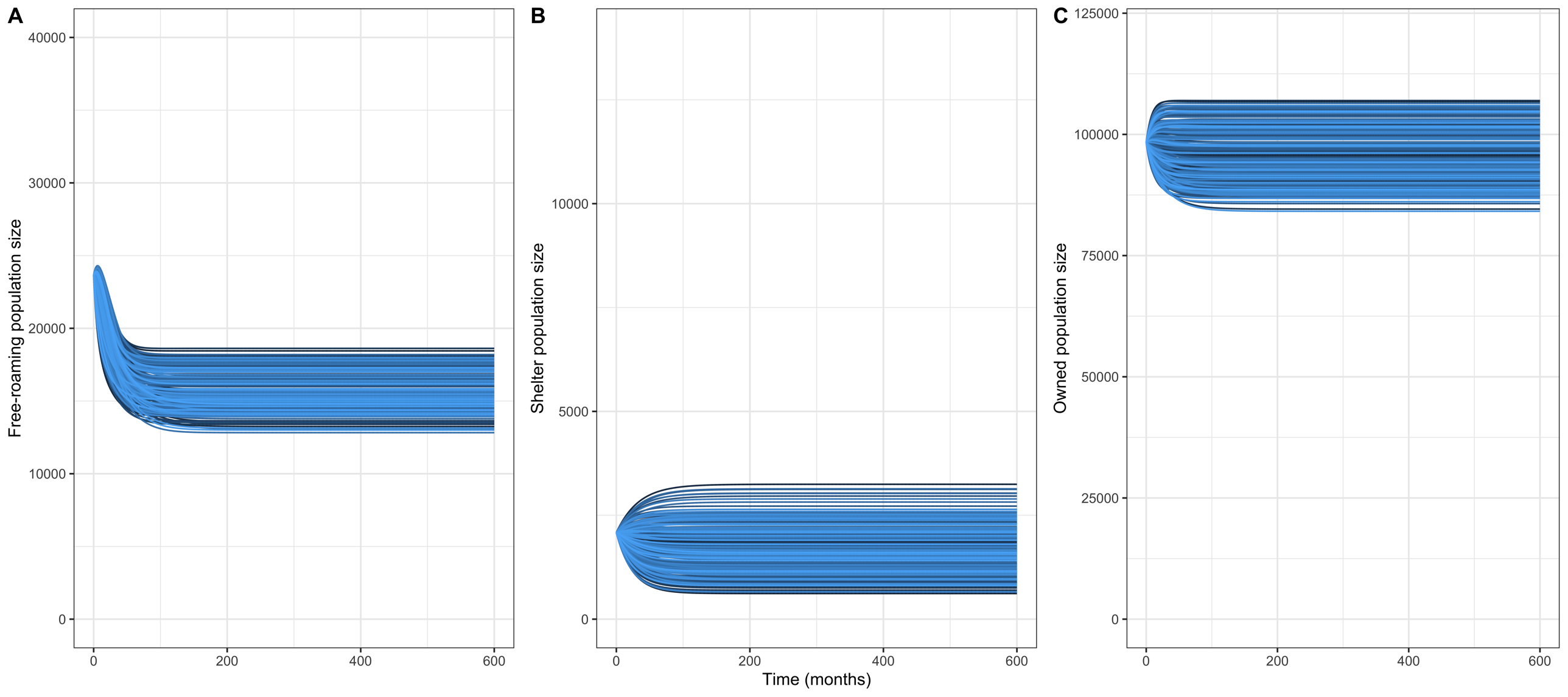


Figure S5. Results of global sensitivity analysis for ***CNR intervention*** on the (A) free-roaming, (B) shelter, and (C) owned dog population sizes.

Simulations generated 100 times and run for 600 months (50-years).


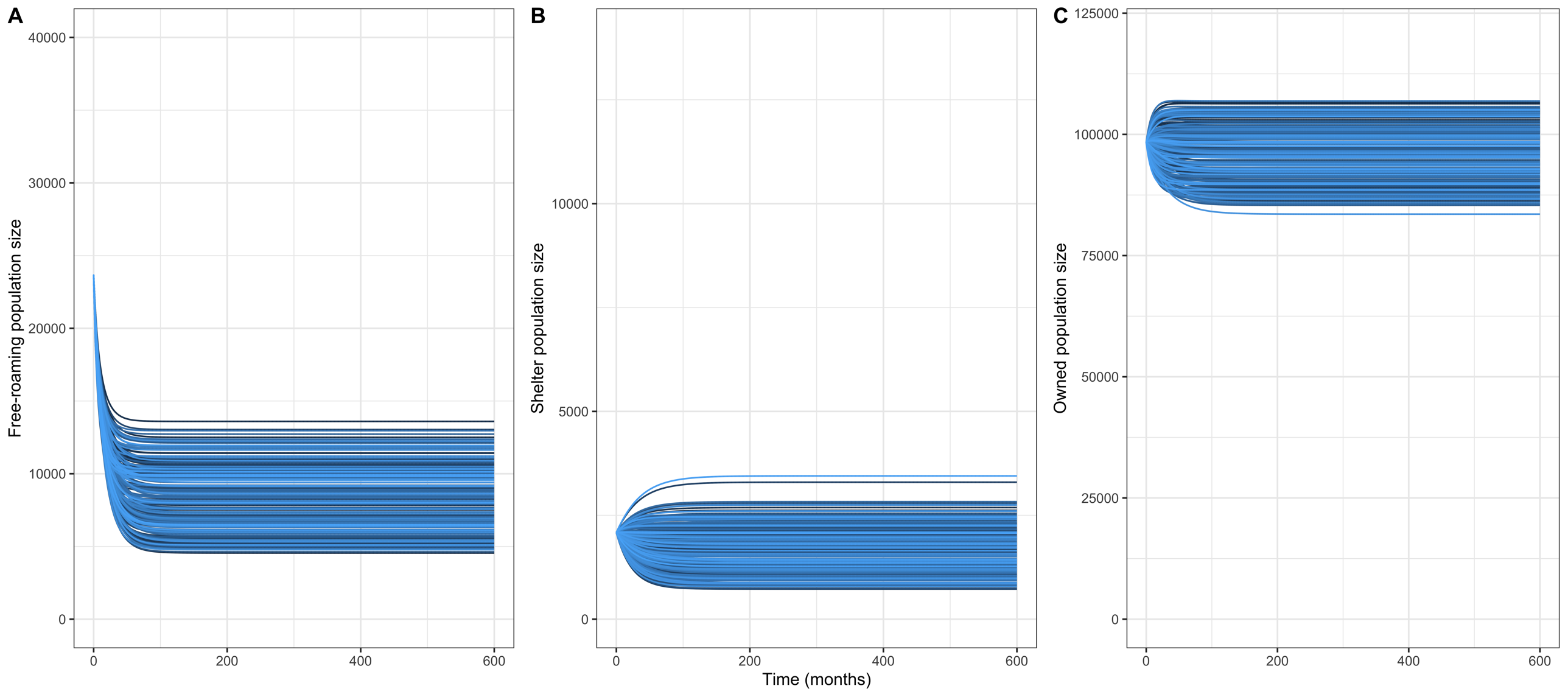


Figure S6. Results of global sensitivity analysis for ***culling intervention*** on the (A) free-roaming, (B) shelter, and (C) owned dog population sizes.

Simulations generated 100 times and run for 600 months (50-years).


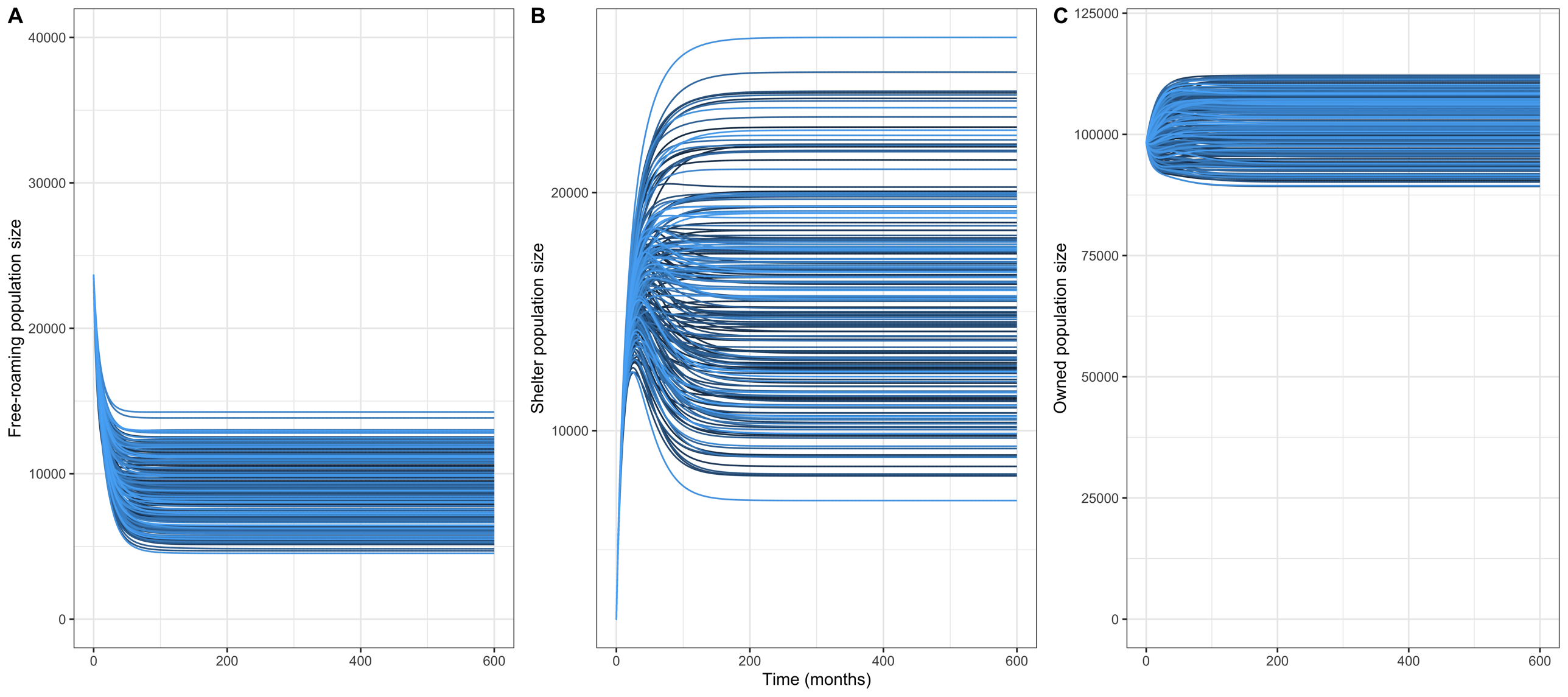


Figure S7. Results of global sensitivity analysis for ***sheltering intervention*** on the (A) free-roaming, (B) shelter, and (C) owned dog population sizes.

Simulations generated 100 times and run for 600 months (50-years).


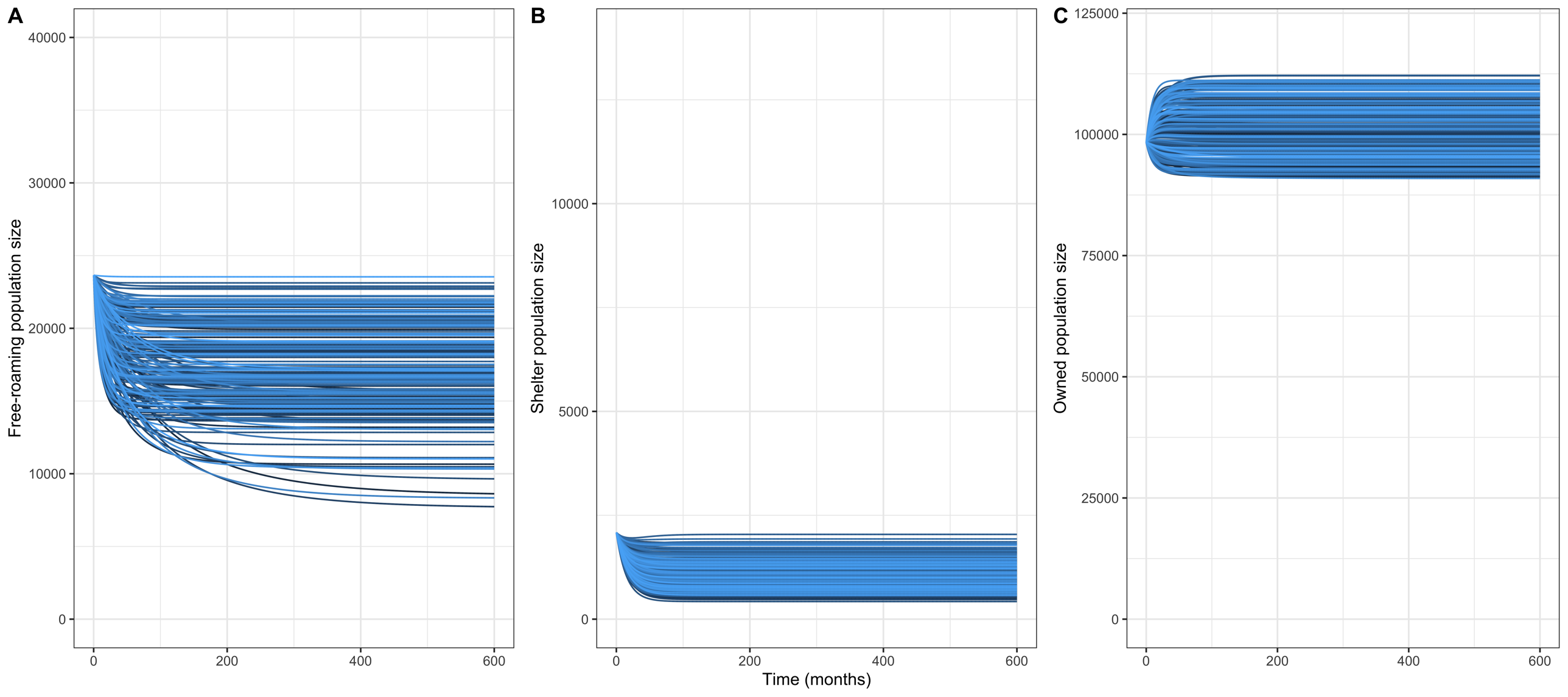


Figure S8. Results of global sensitivity analysis for ***responsible ownership intervention*** on the (A) free-roaming, (B) shelter, and (C) owned dog population sizes.

Simulations generated 100 times and run for 600 months (50-years).


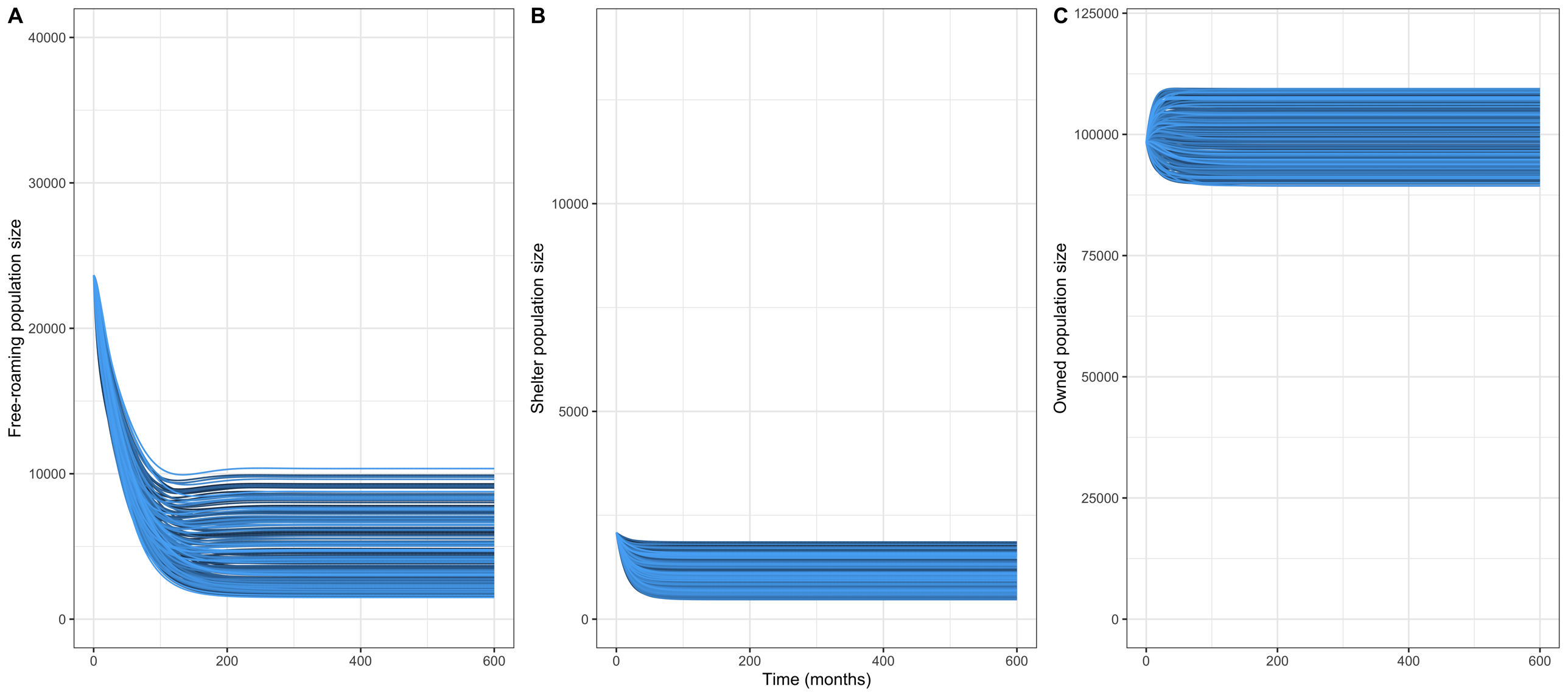


Figure S9. Results of global sensitivity analysis for ***combined CNR and responsible ownership intervention*** on the (A) free-roaming, (B) shelter, and (C) owned dog population sizes.

Simulations generated 100 times and run for 600 months (50-years).


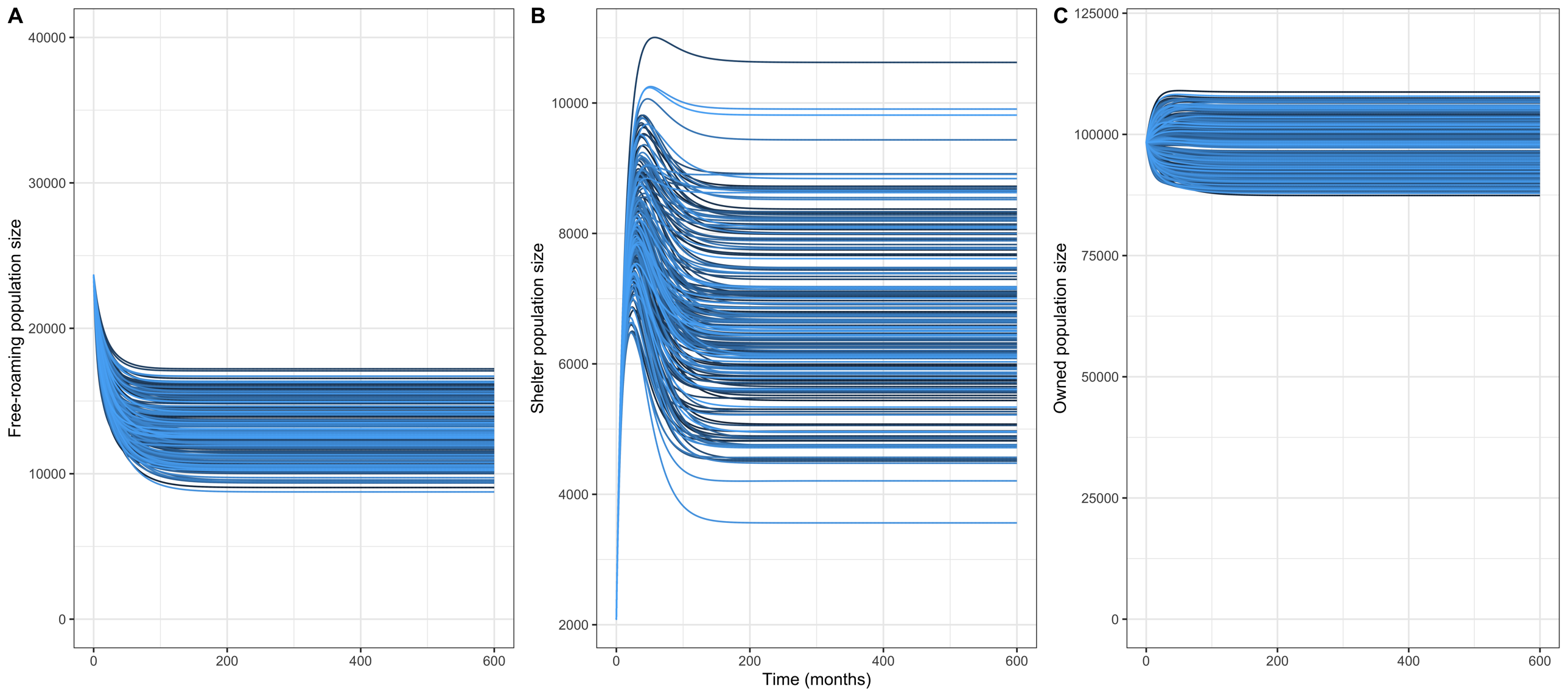


Figure S10. Results of global sensitivity analysis for ***combined CNR and sheltering intervention*** on the (A) free-roaming, (B) shelter, and (C) owned dog population sizes.

Simulations generated 100 times and run for 600 months (50-years).

**References**

1. Smith. The impact of dog population management on free-roaming dog population dynamics, health and welfare. (University of Leeds, 2020).

2. World Organisation for Animal Health(OIE). *Stray dogs population control*. *Terrestrial Animal Health Code, 24th Ed. OIE, Paris* 1–12 (2019).

3. Smith, L. M. *et al.* Attitudes towards free-roaming dogs and dog ownership practices in Bulgaria, Italy, and Ukraine. *PLoS One* **17**, 1–23 (2022).

4. Hsu, Y., Severinghaus, L. L. & Serpell, J. A. Dog Keeping in Taiwan : Its Contribution to the Problem of Free-Roaming. *J. Appl. Anim. Welf. Sci.* **6**, 1–23 (2003).

5. Fielding, W. J. & Plumridge, S. J. Characteristics of owned dogs on the island of New Providence, The Bahamas. *J. Appl. Anim. Welf. Sci.* **8**, 245–260 (2005).

6. Bartlett, P., Bartlett, A., Walshaw, S. & Halstead, S. Rates of euthanasia and adoption for dogs and cats in Michigan animal shelters. *J. Appl. Anim. Welf. Sci.* **8**, 97–104 (2005).

7. Chua, D., Rand, J. & Morton, J. Surrendered and stray dogs in Australia—Estimation of numbers entering municipal pounds, shelters and rescue groups and their outcomes. *Animals* **7**, 1–28 (2017).

8. Hart, L. A., Takayanagi, T. & Yamaguchi, H. Dogs and cats in animal shelters in Japan. *Anthrozoos* **11**, 157–163 (1998).

9. Hemy, M., Rand, J., Morton, J. & Paterson, M. Characteristics and Outcomes of Dogs Admitted into Queensland RSPCA Shelters. *Animals* **7**, 2–23 (2017).

10. Marston, L. C., Bennett, P. C. & Coleman, G. J. What Happens to Shelter Dogs ? An Analysis of Data for 1 Year From Three Australian Shelters. *J. Appl. Anim. Welf. Sci.* **7**, 27–47 (2010).

11. Rinzin, K. *et al.* Free-roaming and surrendered dogs and cats submitted to a humane shelter in Wellington, New Zealand, 1999-2006. *N. Z. Vet. J.* **56**, 297–303 (2008).

12. Talamonti, Z. *et al.* A description of the characteristics of dogs in, and policies of 4 shelters in, different countries. *J. Vet. Behav.* **28**, 25–29 (2018).

13. Amaku, M., Dias, R. A. & Ferreira, F. Dynamics and Control of Stray Dog Populations. *Math. Popul. Stud.* **17**, 69–78 (2010).

14. Carroll, M. J., Singer, A., Smith, G. C., Cowan, D. P. & Massei, G. The use of immunocontraception to improve rabies eradication in urban dog populations. *Wildl. Res.* **37**, 676–687 (2010).

15. Beck, A. *The ecology of stray dogs: a study of free-ranging urban animals*. (1973).

16. Cleaveland, S. & Dye, C. Maintenance of a microparasite infecting several host species: rabies in the Serengeti. *Parasitology* **111**, S33–S47 (1995).

17. Skalski, J., Ryding, K. & Millspaugh, J. *Wildlife Demography: Analysis of Sex, Age and Count Data*. (Elsevier Academic Press, 2005).

18. Lotka, A. . *Elements of Physical Biology*. (Williams and Wilkins, 1925).

19. Cole, L. C. The Population Consequences of Life History Phenomena. *Q. Rev. Biol.* **29**, 103–137 (1954).

20. Caughley, G. *The analysis of vertebrate populations*. (John Wiley & Sons, 1977).

21. Acosta-Jamett, G., Cleaveland, S., Cunningham, A. A. & Bronsvoort, B. M. deC. Demography of domestic dogs in rural and urban areas of the Coquimbo region of Chile and implications for disease transmission. *Prev. Vet. Med.* **94**, 272–281 (2010).

22. Costa, E. D. *et al.* Impact of a 3-year pet management program on pet population and owner’s perception. *Prev. Vet. Med.* **139**, 33–41 (2017).

23. New, J. *et al.* Birth and death rate estimates of cats and dogs in US households and related factors. *J. Appl. Anim. Welf. Sci.* **7**, 229–241 (2004).

24. Greer, K. A., Canterberry, S. C. & Murphy, K. E. Statistical analysis regarding the effects of height and weight on life span of the domestic dog. *Res. Vet. Sci.* **82**, 208–214 (2007).

25. Czupryna, A. *et al.* Ecology and Demography of Free-Roaming Domestic Dogs in Rural Villages near Serengeti National Park in Tanzania. *PLoS One* **11**, (2016).

26. Hampson, K. *et al.* Transmission dynamics and prospects for the elimination of canine rabies. *PLoS Biol* **7**, 462–471 (2009).
